# Tuning the Lifetimes of Photoinduced Deligation in a Metal–Organic Framework via Linker Functionalization

**DOI:** 10.1021/jacs.5c09921

**Published:** 2025-09-10

**Authors:** Qingyu Ye, Isabelle A. Herlinger, Lisa A. Fredin, Daniel R. Cairnie, Xiaozhou Yang, Minliang Yan, Amanda J. Morris

**Affiliations:** † Department of Chemistry, Virginia Tech, Blacksburg, Virginia 24060, United States; ‡ Department of Chemistry, 1687 Lehigh University, Bethlehem, Pennsylvania 18015, United States

## Abstract

Recently photoinduced dynamic ligation in a metal–organic frameworks (MOFs) was reported, where a long-lived charge-transfer excited state (ca. 30 μs) featuring partial dissociation between the carboxylate linker and metal-based node was probed by time-resolved infrared (TRIR) spectroscopy. The study offers a new mechanistic perspective to evaluate the potential contribution from the excited state molecular configuration to the performance of MOF photocatalysts. In this work, by employing MIL-101­(Fe) as the study platform, we have further explored the influence of intramolecular interactions on the stability of relevant excited states and demonstrated the effective tuning of their lifetimes through the incorporation of different functional groups into the system. The correlations between the varied excited state lifetimes and coordination configurations with specific functional groups (−NH_2_ or −NO_2_) was inferred from the analyses of infrared spectroscopic data and theoretical calculations, revealing the essential role of the intramolecular interactions (i.e., between the added functional groups and the carboxylate group) in the modulation of system energetics. Overall, the work presents a pathway to tune the excited state dynamics and expands the knowledge regarding the photoinduced dynamic ligation in carboxylate-based MOFs.

## Graphical abstract

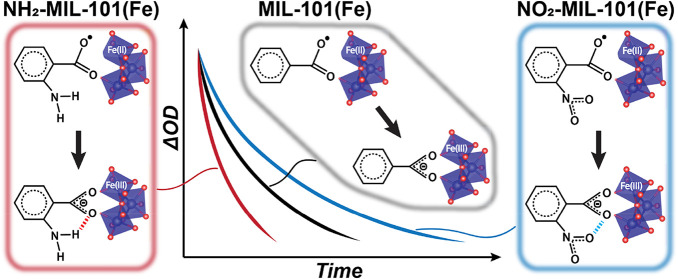

## Introduction

By promoting reactions with light, photocatalysis grants us the capacity to harness abundant solar energy to produce desired chemicals, e.g., the use of photoactivated ruthenium polypyridyl complexes to catalyze carbon dioxide reduction.^1–3^ However, such molecular photocatalysts often suffer from photoinduced degradation, including dissociation and dimerization, undermining their overall performances.^4–6^

One potential strategy to mitigate the dissociation and dimerization effects is to spatially isolate the photocatalysts and restrict them to fewer degrees of freedom by incorporating and/or encapsulating them into a molecular matrix. In this regard, metal organic frameworks (MOFs) represent a promising architecture with demonstrated capability to integrate various catalytic moieties, thanks to their well-defined crystalline and porous structures with outstanding molecular level tunability. Moreover, granted with an exceedingly high surface-to-volume ratio, MOFs are generally considered as the promising candidate for novel heterogeneous catalysis, which is a rapidly expanding field.^7^

Indeed, MOFs featuring open metal sites have been widely explored as photocatalysts.^8–10^ While these open-metal sites were often accounted for from a configurationally static model, we have recently revealed that they might be transiently generated upon photoexcitation.^11^ Specifically, a photoinduced reversible transition between different coordination modes of a carboxylate MOF [MIL-101­(Fe)] was characterized. Compared to the bidentate ground state, the photoexcited states featured an asymmetrical monodentate configuration with ligand-to-metal charge transfer (LMCT) character, indicating a partial dissociation of the carboxylate ligand from one of the nodal Fe atoms. The probed transient deligated state exhibited a lifetime of ca. 30 μs that is of catalytic interest since it significantly exceeds the temporal threshold posed by bimolecular diffusional kinetics (i.e., few ns). A similar phenomenon was later observed in a Ti-carboxylate MOF system.^12^ The observed phenomenon offers a new angle in understanding some MOF photocatalysts, as the dynamic ligation itself might be a contributor to the overall photocatalysis performance.

Given the implications of the photoinduced dynamic ligation, it would be beneficial to further uncover the factors governing the stability of the relevant transient states in MOFs so that the effective tuning of their lifetimes can be realized. Fortunately, with the synthetic tunability of MOFs, including modifications to both the constituent linkers and metal-based nodes (e.g., the well-developed MIL-101 series), we can evaluate the coordination lability as a function of various parameters, including (but not limited to) intramolecular interactions, bonding strength, framework geometry, and the electronic configuration of the metal nodes.

Herein, we carried out a spectroscopic investigation into the stability of the photoinduced deligation states in a series of MIL-101­(Fe) MOFs, i.e., MIL-101­(Fe), NH_2_-MIL-101­(Fe), and NO_2_-MIL-101­(Fe). By jointly probing the electronic and vibrational dynamics of the three photoexcited MOFs through visible transient absorption (VisTA) and time-resolved infrared (TRIR) spectroscopy, the effective modulation of the transient deligation state lifetimes upon the addition of either −NH_2_ or −NO_2_ to the system was confirmed. The kinetic analysis of the TRIR data resolved species corresponding to different coordination configurations in the systems. Together with a closer evaluation of the FTIR spectra and computational support, the varied lifetimes of these excited species were attributed to the interactions between the added functional groups and the carboxylate group in the MOFs. The validity of the mechanism was further justified by the characterizations of another MIL-101 derivative, OH-MIL-101­(Fe). The work demonstrates an efficient strategy to tune the lifetimes of the photoinduced deligation in MIL-101 series and provides a perspective to comprehend the dynamic ligation in carboxylate MOFs.

## Results and Discussion

Following a similar procedure (see Supporting Information (SI) for details), three MOFs with varied linkers were synthesized, MIL-101­(Fe), NH_2_-MIL-101­(Fe) and NO_2_-MIL-101­(Fe) (Figure 1). The power X-ray diffraction (PXRD) patterns of the MOFs match that of the simulated MIL-101 structure (Figure S1).^13^ The typical octahedral crystal shape of MIL-101 was also confirmed by scanning electron microscope (SEM) (Figures S2–S4).^14^

**Figure 1:**
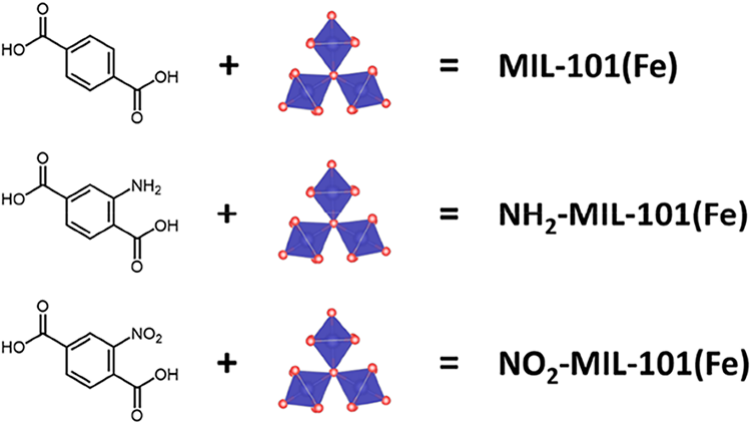
Schematic illustrations for the constituents of MIL-101­(Fe), NH_2_-MIL-101­(Fe) and NO_2_-MIL-101­(Fe). The respective linker molecules used for synthesis are benzene-1,4-dicarboxylic acid (BDC), 2-aminobenzene-1,4-dicarboxylic acid (NH_2_–BDC) and 2-nitrobenzene-1,4-dicarboxylic acid (NO_2_–BDC). The Fe-μ_3_-oxo cluster serves as the MOF node.

Ground state electronic absorption features of the MOFs and their linker molecules were acquired with the diffuse reflectance mode of the spectrometer, as shown in Figure 2a–c. The spectra of the linkers shared a strong absorption peak at 226 nm, corresponding to π–π* transitions. Meanwhile, the n−π* features displayed slightly different spectral profiles for different linkers, which reflected the modulation of the nonbonding orbital energies by the additional functional groups. Specifically, besides the absorptive region peaking at 300 nm for BDC, new absorptions centered around 394 and 350 nm for NH_2_–BDC and NO_2_–BDC were present, respectively. For the MOFs, while the π–π* peaks were mostly present with slight red shift due to the formation of more delocalized structures, new strong bands emerged in addition to the ligand-based transitions (the estimated ranges are marked as shaded regions), which were indicative of LMCT transitions introduced by MOF node-linker coordination.^8,11,15^ As the partial ligand dissociation is linked to LMCT events, the spectral ranges of these bands suggest the optimal excitation wavelengths to be used for the time-resolved investigations.

**Figure 2:**
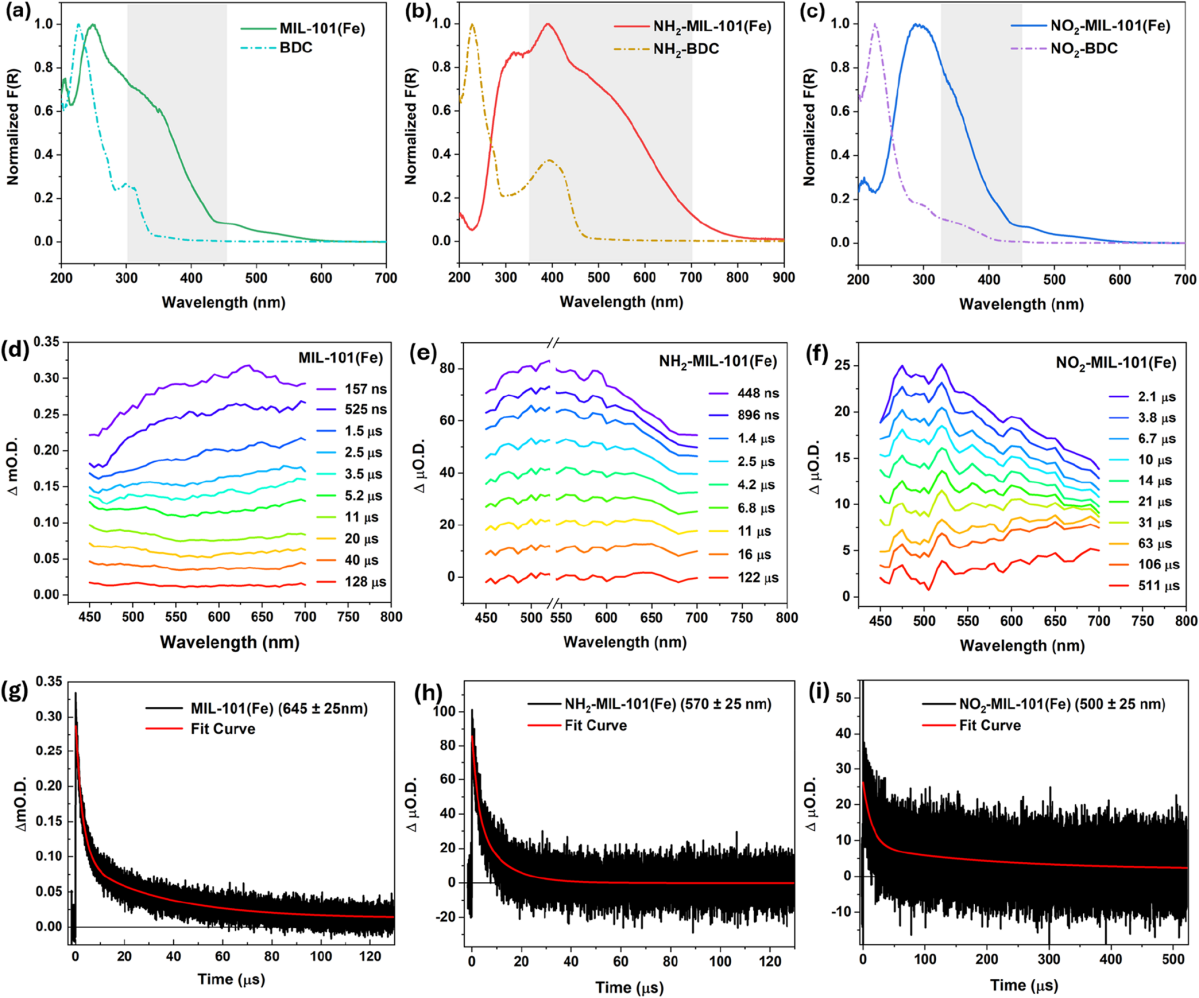
UV–vis electronic spectroscopic characterizations of MIL-101­(Fe), NH_2_-MIL-101­(Fe), and NO_2_-MIL-101­(Fe) from left to right. (a, b, c) Ground-state absorption spectra acquired via diffuse reflectance for the MOFs and their corresponding BDC linker molecules. The shaded regions mark the estimated spectral ranges associated with LMCT transitions in the MOFs. (d, e, f) VisTA spectra of the three MOFs at varied time delays upon 355, 532 and 355 nm excitation, respectively. (g, h, i) Selected kinetic traces of MOFs at 645 ± 25 nm, 570 ± 25 nm and 500 ± 25 nm (black) and their corresponding biexponential fits (red).

To probe the electronic excited states primarily associated with LMCT, VisTA spectroscopy was first conducted for MIL-101­(Fe), NH_2_-MIL-101­(Fe) and NO_2_-MIL-101­(Fe) with 355, 532 and 355 nm excitations, respectively. The transient absorption spectra and corresponding kinetic traces of the MOFs are shown in Figure 2d–i. All three samples exhibited broad excited state absorptions (ESA) across the 450–700 nm detected range, reflecting the rich and complex electronic configurations of these photoexcited systems, which suggested the delocalized character and the removed degeneracy of the Fe d orbital after LMCT. The further removal of the d orbital degeneracy resulted from the decreased ligand field symmetry upon the transient ligand dissociation. Note that multiple excited states could be identified by comparing the time-dependent TA map in different spectral ranges. Kinetic analysis at the spectral regions with maximum TA signals revealed lifetimes of these major species in the photoexcited MOFs. Specifically, MIL-101­(Fe) displayed a biexponential decay at 645 ± 25 nm, with average lifetimes of 3.25 ± 0.03 μs and 34.2 ± 0.2 μs. For NH_2_-MIL-101­(Fe) at 570 ± 25 nm and NO_2_-MIL-101­(Fe) at 500 ± 25 nm, two-component decay traces with time constants of 2.5 ± 0.2/11.0 ± 0.5 μs and 14.2 ± 0.4/200 ± 10 μs were recorded, respectively. The fitted lifetimes are summarized in Table 1. Notably, the lifetimes of these excited species were significantly modulated in the presence of the (different) functional groups.

**Table 1:** Fitted Time Constants for MIL-101­(Fe), NH_2_-MIL-101­(Fe) and NO_2_-MIL-101­(Fe) Based on VisTA Measurements

	MIL-101(Fe) @645 ± 25 nm	NH_2_-MIL-101(Fe) @570 ± 25 nm	NO_2_-MIL-101(Fe) @500 ± 25 nm
τ_1_ (μs)	3.25 ± 0.03	2.5 ± 0.2	14.2 ± 0.4
τ_2_ (μs)	34.2 ± 0.2	11.0 ± 0.5	200 ± 10

The ground-state vibrational spectra were then investigated (Figure 3a–c). The detailed spectral positions of relevant peaks are summarized in Table 2. In all three MOFs, there were general features identified at specific spectral ranges. These mainly include the characteristic symmetric [ν_sym_(COO^–^)]/asymmetric [ν_as_(COO^–^)] carboxylate stretches and the two major aromatic ring stretches [ν­(C_ar_–C_ar_)], i.e., 1387/1608 cm^–1^ and 1431/1506 cm^–1^ for MIL-101­(Fe), 1383/1617 cm^–1^ and 1428/1495 cm^–1^ for NH_2_-MIL-101­(Fe), 1387/1627 cm^–1^ and 1404/1496 cm^–1^ for NO_2_-MIL-101­(Fe).^11,16^ Additionally, two minor peaks at around 1160/1660 cm^–1^ suggested the traces of C–O/C=O stretches, which could be attributed to residue BDC linkers and/or missing node defects in the MOFs. Compared to MIL-101­(Fe), more vibrational modes were present in the functionalized MOFs.^16^ For NH_2_-MIL-101­(Fe), in addition to the C–N stretch at 1256 cm^–1^,^17^ there were more peaks (at 1336 and 1577 cm^–1^) on the red side of the corresponding generic carboxylate stretches, which suggested the influence of the −NH_2_ group on the carboxylate modes (vide infra). For NO_2_-MIL-101­(Fe), peaks corresponding to ν­(C–N) and ν_as_(NO_2_) were identified at 1254 and 1544 cm^–1^, respectively.^17^ Considering the symmetric nature of −NO_2_ and the clear presence of both ν_as_(NO_2_) and ν_sym_(NO_2_) peaks in the NO_2_–BDC linker absorption spectrum (Figure S5), the ν_sym_(NO_2_) mode of the MOF was most likely to overlap with the strong ν_sym_(COO^–^) peak around 1387 cm^–1^.^17^

**Figure 3:**
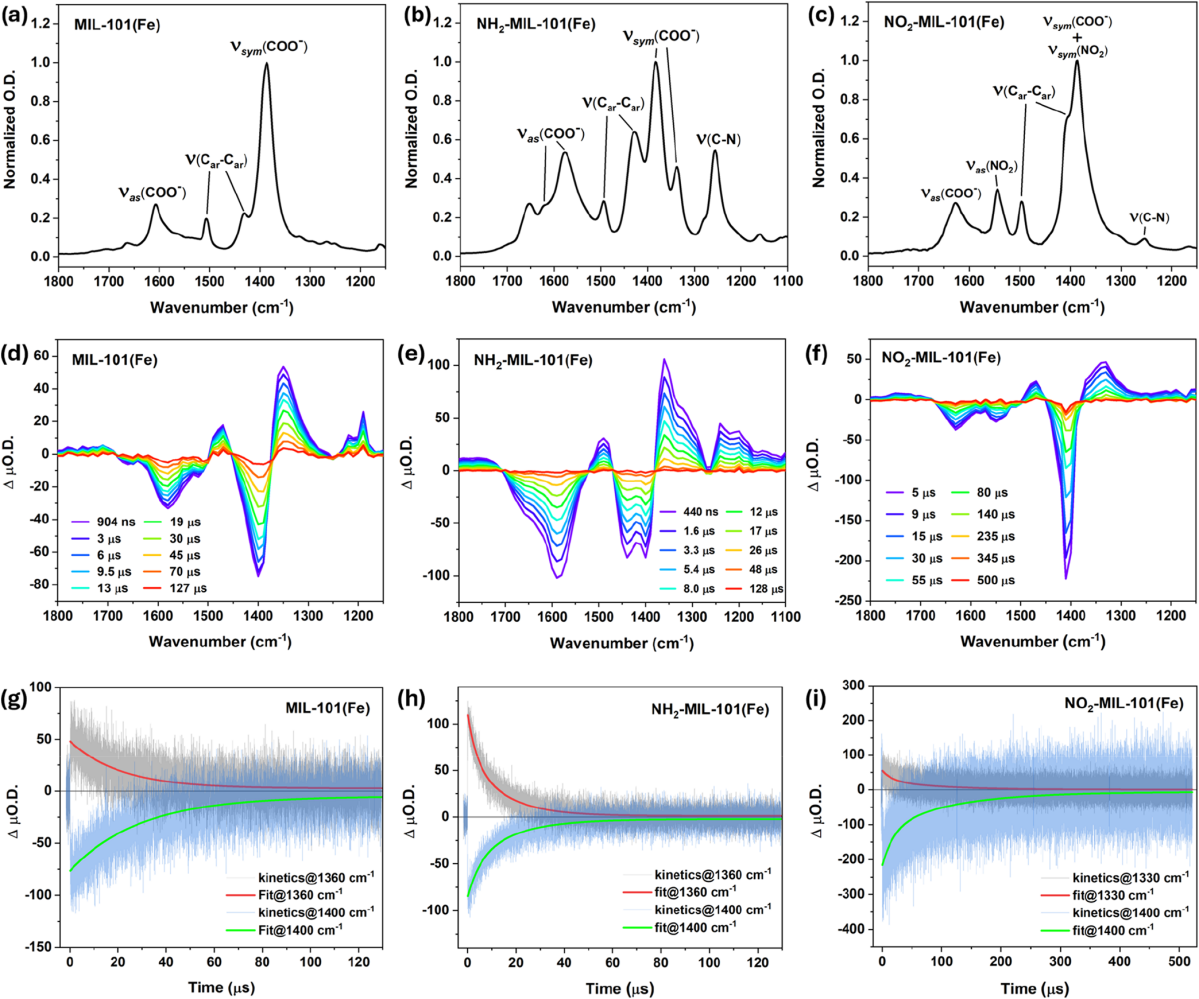
Midinfrared vibrational spectroscopic characterizations of MIL-101­(Fe), NH_2_-MIL-101­(Fe) and NO_2_-MIL-101­(Fe) from left to right. (a, b, c) Ground-state FTIR absorption spectra of the MOFs. Major absorptive peaks are labeled accordingly. (d, e, f) TRIR difference absorption spectra at varied time delays for the MOFs upon 355, 532, and 355 nm excitation, respectively. (g, h, i) Kinetic traces of the MOFs probed at representative wavenumbers (gray and blue) and their corresponding exponential fits (red and green).

**Table 2:** Spectral Positions Corresponding to Major Absorptive Vibrational Modes Displayed in the FTIR Spectra of the MOFs (Unit: cm^–1^)

	MIL-101(Fe)	NH_2_-MIL-101(Fe)	NO_2_-MIL-101(Fe)
ν_sym_(COO^–^)/ν_as_(COO^–^)	1387/1608	1383/1617 (generic)	1387/1627
		1336/1577 (−NH_2_ modulated)	
ν(C_ar_–C_ar_)	1431/1506	1428/1495	1404/1496
ν(C–N)	-	1256	1254
ν_sym_(NO_2_)/ν_as_(NO_2_)	-	-	1387/1544
residue linker and/or defect ν(C–O)/ν(C=O)	1161/1664	1160/1652	1166/-^a^

a The exact spectral position is hard to distinguish due to its weak absorption and the overlap with ν_as_(COO^–^).

The bonding dynamics of the photoexcited MOFs were measured via TRIR spectroscopy, by applying the same pump wavelengths used for VisTA measurements. In general, the transient difference absorbance spectra of the three MOFs shared a similar pattern to that reported previously (Figure 3d–f).^11^ First, ground-state bleaches (GSB) corresponding to the major ground-state absorptions were present with clear local minima, i.e., 1400 cm^–1^ [ν_sym_(COO^–^)] and 1580 cm^–1^ [ν_as_(COO^–^)] for MIL-101­(Fe), 1400 cm^–1^ [ν_sym_(COO^–^)], 1440 cm^–1^ [ν­(C_ar_–C_ar_)] and 1590 cm^–1^ [ν_as_(COO^–^)] for NH_2_-MIL-101­(Fe), 1410 cm^–1^ [ν_sym_(COO^–^)], 1550 cm^–1^ [ν_as_(NO_2_)] and 1630 cm^–1^ [ν_as_(COO^–^)] for NO_2_-MIL-101­(Fe). Second, two ESA features emerged from the far red and blue side of the measured spectral range [peak at 1190/1710 cm^–1^, 1200/1760 cm^–1^ and 1190/1750 cm^–1^ for MIL-101­(Fe), NH_2_-MIL-101­(Fe) and NO_2_-MIL-101­(Fe), respectively], representing the loss of ground state symmetry and the formation of C–O and C=O bonds on the photoexcited states in all MOFs. Third, ESAs associated with the Franck–Condon states^18^ generated from the back electron transfers could be found on the red side of major GSBs, reflecting the anharmonicity of the ground-state oscillators. According to the previous study,^11,19^ the common presence of the second character indicated that the photoinduced partial ligand dissociation was an event occurred in all three systems.

Kinetic analysis at major transient peak positions provided lifetimes of the excited states. For MIL-101­(Fe), monoexponential decays with lifetimes of 21.0 ± 0.5 μs and 28.8 ± 0.6 μs at 1360 and 1400 cm^–1^ respectively are shown in Figure 3g. On the other hand, the kinetics of NH_2_-MIL-11­(Fe) and NO_2_-MIL-101­(Fe) could be best fitted by a biexponential model with components featuring distinct time constants [Figure 3h,i]. The fitted lifetimes of the three MOFs at representative wavenumbers are presented in Table 3. Comparing the components resolved from TRIR and VisTA measurements, specific consistency in their time scales could be noticed. Namely, the TRIR lifetime was close to the longer component from VisTA for MIL-101­(Fe), and the shorter/longer components from TRIR were comparable to their counterparts from VisTA for the functionalized MOFs. These suggested that the excited-state dynamics of the same events were probed from both the vibrational and the electronic aspects of the systems. Note that, compared to the VisTA kinetics, the missing of a shorter component in MIL-101­(Fe) from TRIR measurement implicated that the shorter-lived electronic excited state observed in VisTA did not induce a significant modulation of the vibrational modes of the MOF.

**Table 3:** Fitted Time Constants for MIL-101­(Fe), NH_2_-MIL-101­(Fe) and NO_2_-MIL-101­(Fe) Based on TRIR Measurements

	MIL-101(Fe)		NH_2_-MIL-101(Fe)		NO_2_-MIL-101(Fe)	
wavenumber (cm^–1^)	1360	1400	1360	1400	1330	1400
τ_1_ (μs)	21.0 ± 0.5	28.8 ± 0.6	3.5 ± 0.3	5.2 ± 0.6	15.8 ± 1.7	19.7 ± 1.4
τ_2_ (μs)	-	-	14.4 ± 0.4	17.7 ± 1.0	90.2 ± 5.4	113.9 ± 5.3

Intriguingly, the lifetimes of the longer component of NH_2_-MIL-101­(Fe) and the shorter component of NO_2_-MIL-101­(Fe) were in very good agreement with each other, and close in value to the single lifetime observed for MIL-101­(Fe), which indicated that these species could originate from a similar molecular configuration. Indeed, given the asymmetric functionalization of the employed BDC ligands, molecular configurations of the generic COO^–^ without adjacent functional group like that presented in MIL-101­(Fe) and the COO^–^ with an adjacent functional group coexisted in NH_2_-MIL-101­(Fe) and NO_2_-MIL-101­(Fe). Therefore, we postulated that the consistent lifetime over all MOFs corresponded to the photoinduced deligation of the carboxylate moiety without an adjacent functional group. Accordingly, the presence of an additional (shorter or longer) component in TRIR kinetics of the functionalized MOFs should correlate to the deligation modulated through the presence of adjacent functional groups (−NH_2_ or −NO_2_). It is noteworthy that the addition of these functional groups could drastically tune the lifetimes of the photoinduced deligation events by 2 orders of magnitude.

A closer examination of the FTIR spectra verifies the presence of multiple species and intramolecular interactions in the functionalized MOFs. Specifically, in addition to the carboxylate modes [ν_sym_(COO^–^) at 1383 cm^–1^ and ν_sym_(COO^–^) at 1617 cm^–1^] that were also present in MIL-101­(Fe), NH_2_-MIL-101­(Fe) featured strong peaks on the red side of these generic carboxylate peaks, i.e., at 1336 and 1577 cm^–1^. These peaks reflected the modulation of the COO^–^ vibrational modes due to the presence of −NH_2_, indicating an interaction between the two constituents (COO^–^ and −NH_2_). The interaction was confirmed by comparing the fingerprint modes of −NH_2_ [i.e., ν_sym_(NH_2_) and ν_as_(NH_2_)] in the NH_2_–BDC molecule and in the MOF, where significant red shifts of the corresponding peaks were observed upon the formation of NH_2_-MIL-101­(Fe) (Figure 4 left).^17^ The red shifts corresponded to the weakening of the N–H bonds in −NH_2_, which strongly indicated an attractive interaction (hydrogen bonding)^20^ between the positively charged hydrogen atoms from −NH_2_ and the nearby negatively charged oxygen atom of the carboxylate. Likewise, though no additional strong absorption peaks could be clearly marked out in the FTIR spectrum of NO_2_-MIL-101­(Fe), the blue shifts of the −NO_2_ peaks [ν_sym_(NO_2_) and ν_as_(NO_2_) modes] upon the MOF formation (Figure 4 right) evidenced the repulsive interaction between the negatively charged oxygens from −NO_2_ and COO^–^.

**Figure 4:**
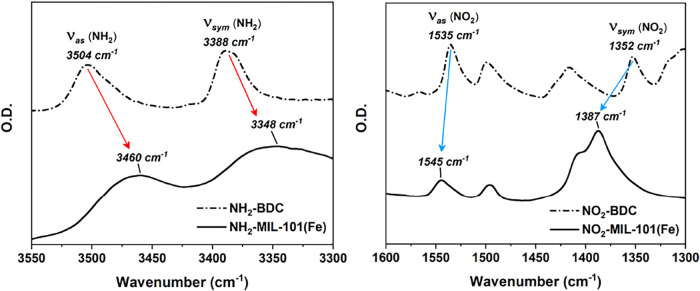
FTIR spectra displaying the characteristic vibrational modes of −NH_2_ and −NO_2_ groups in BDC linkers and in the respective MOFs. (left) upon the formation of NH_2_-MIL-101­(Fe), ν_sym_(NH_2_) and ν_as_(NH_2_) red-shifted from 3388 cm^–1^ and 3504 cm^–1^ to 3348 cm^–1^ and 3460 cm^–1^, indicating the weakening of the N–H bonds. (right) upon the formation of NO_2_-MIL-101­(Fe), ν_sym_(NO_2_) and ν_as_(NO_2_) blue-shifted from 1352 cm^–1^ and 1535 cm^–1^ to 1387 cm^–1^ and 1545 cm^–1^, indicating the strengthening of the N–O bonds.

Theoretical calculations also support the existence of the attractive or repulsive interactions due to the introduction of different functional groups (see SI for details). In brief, the average distance between the functional group and closest carboxylate oxygen in NH_2_-MIL-101­(Fe) appears to be much shorter than that in NO_2_-MIL-101­(Fe) (e.g., 1.918 Å as compared to 2.772 Å in closed shell model; Table S1, Figure S9). Moreover, the ligand-node binding energies of the three tested systems exhibit an order with increased stability: NO_2_-MIL-101­(Fe), MIL-101­(Fe), NH_2_-MIL-101­(Fe) (Table S2).

Given the spectroscopic evidence and computational corroboration of the interactions between the functional groups and carboxylates in the MOFs, we reason the influence of the functional groups on the stability of the photoinduced transient deligation states in a qualitative manner. For the shorter-lived excited state component of NH_2_-MIL-101­(Fe), the shorter lifetime indicated a lower free energy barrier (Δ*G* ^⧧^) for back electron transfer (BET) in the presence of −NH_2_. Moreover, the attractive interaction between −NH_2_ and COO^–^ lowers the ground state energy. Since the oxygen atom from the original carboxylate should be less charged in the excited state (given its LMCT character), it is assumed that the excited state energy was not altered as much as the ground state due to the weaker interaction with −NH_2_. Therefore, there was an increase in the driving free energy (Δ*G°*) of BET for photoexcited NH_2_-MIL-101­(Fe) along with the decrease of the free energy barrier, which means that the BET from the charge-separated state belongs to a regime resembling the Marcus normal region (Figure 5). Similarly, the longer-lived species in the photoexcited NO_2_-MIL-101­(Fe) was associated with the higher BET energy barrier accompanied by a net decrease of driving free energy, due to the repulsive interaction between −NO_2_ and COO^–^ on the ground state (Figure 5).

**Figure 5:**
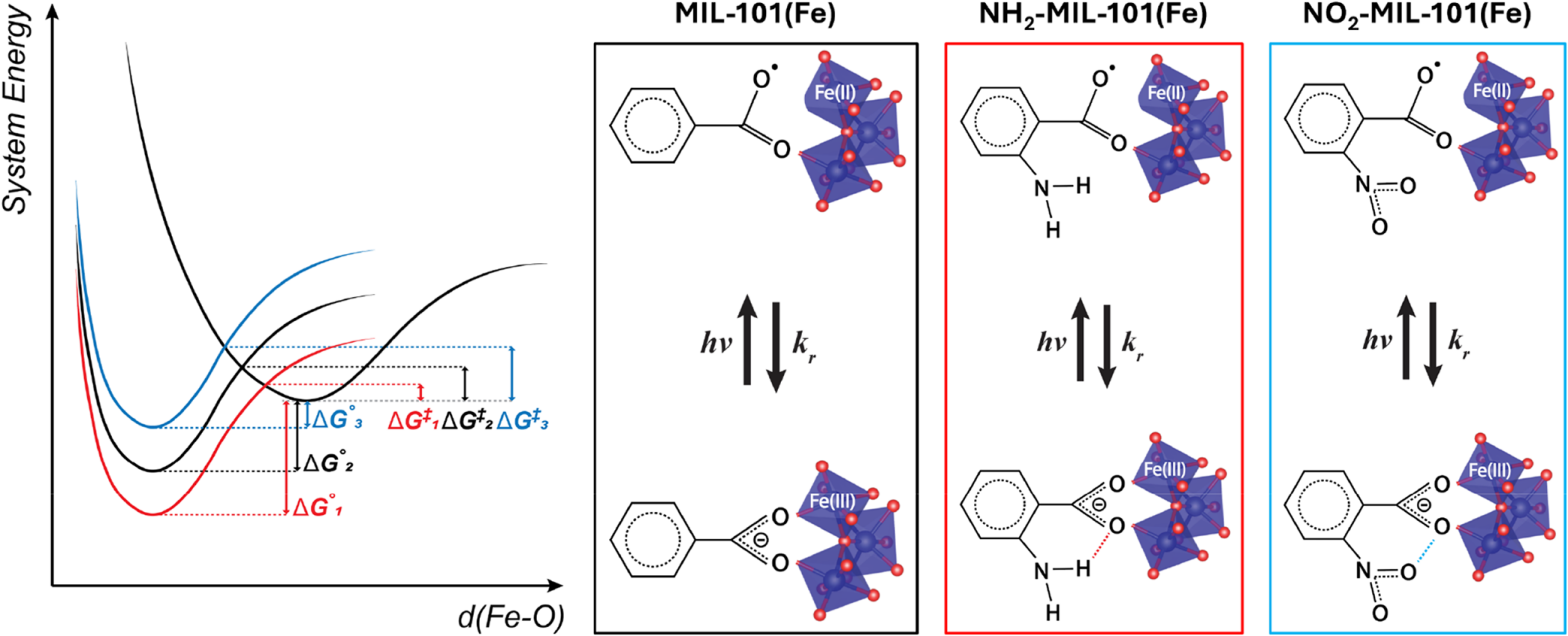
Mechanistic schemes comparing the photoinduced ligand dissociation events in the three MOFs [parameters of MIL-101­(Fe), NH_2_-MIL-101­(Fe) and NO_2_-MIL-101­(Fe) are marked respectively with footnote number/color of “2”/black, “1”/red and “3”/blue]. The free energy barrier (Δ*G* ^⧧^) restricting the back electron transfer (BET) could be modulated by the interactions between added functional groups and the nearby carboxylate. Note that due to the LMCT character of the transition, the associated carboxylate oxygen should be less charged on the excited state, and the above-mentioned interactions would be weakened compared to the ground state. The comparably weaker modulation of the excited state energy is represented by a shared excited-state surface for all three MOFs and the scheme emphasizes the net change of the BET driving free energy Δ*G*° (and Δ*G* ^⧧^) with the presence of functional groups. In the boxed schemes, the attractive interaction between −NH_2_ and COO^–^ in NH_2_-MIL-101­(Fe) is marked as red dashed line and the repulsive interaction between −NO_2_ and COO^–^ in NO_2_-MIL-101­(Fe) is marked as blue dashed line.

According to the model proposed, we should be able to predict the excited state behaviors of similar ligand modifications if the type of interaction between the additional functional group and COO^–^ is known, or vice versa, i.e., we could tell the presence and type of interaction (attractive or repulsive) between the functional group and COO^–^ from their excited state behaviors. The mechanism was corroborated by the spectroscopic characterizations of OH-MIL-101­(Fe), where OH-BDC was used as the linker molecule instead of BDC (PXRD, SEM, and FTIR characterizations are shown in Figures S6–S8). Like the case of NH_2_-MIL-101­(Fe), the positively charged hydrogen atom of −OH would attractively interact with the negatively charged oxygen atom from COO^–^, which should lead to the generation of an additional short-lived species in the photoexcited MOF. The prediction was confirmed. In brief, at 1430 cm^–1^ where the maximum GSB was recorded, the photoexcited OH-MIL-101­(Fe) exhibited a biexponential TRIR decay kinetics with the longer time constant of 20.0 ± 2.7 μs which was close to that of MIL-101­(Fe) and the shorter one of 7.0 ± 0.7 μs which was indicative of the attractive interaction between −OH and COO^–^ in the MOF (Figure S10).

## Conclusions

In summary, we have characterized the ground/excited state features of a MOF series [MIL-101­(Fe), NH_2_-MIL-101­(Fe), NO_2_-MIL-101­(Fe)] and demonstrated the effective regulation of the photoinduced deligation lifetimes in these systems. The excited state dynamics of the MOFs were probed from both the electronic and vibrational aspects via the application of VisTA and TRIR spectroscopy, where an additional shorter- or longer-lived species in the presence of −NH_2_ or −NO_2_ was identified. With the incorporation of these functional groups, the excited-state time constants of the MOFs could be modulated in a range spanning nearly 3 orders of magnitude. Based on detailed spectroscopic analysis and theoretical calculations, the modulation in lifetime was attributed to the Coulomb interactions between the functional groups and the carboxylate group within the frameworks. The proposed model was corroborated by successfully predicting the excited state behavior of OH-MIL-101­(Fe). In general, the work presents a pathway to tune the excited state dynamics and marks one step further toward a better understanding of the photoinduced dynamic ligation in carboxylate-based MOFs.

## Supplementary Material



## Notes

Experimental materials and methods; synthesis procedures; powder diffraction crystallography; scanning electron microscopy; computational methods and results (PDF)
