# Supplementary material for: Tuning the Lifetimes of Photoinduced Deligation in a Metal–Organic Framework via Linker Functionalization
Source: J Am Chem Soc. 2025 Sep 10;147(38):34690–6. doi: 10.1021/jacs.5c09921 (PMC12464993; doi:10.1021/jacs.5c09921)
Supplement: Supplementary file 1 [file ja5c09921_si_001.pdf]

Supporting Information

Tuning the Lifetimes of Photoinduced De-ligation in a Metal-Organic Framework via Linker Functionalization

Qingyu Ye[1], Isabelle A. Herlinger[2], Lisa A. Fredin[2], Daniel R. Cairnie[1], Xiaozhou Yang[1], Minliang Yan[1], Amanda J. Morris[1]*

1. *Department of Chemistry, Virginia Tech, Blacksburg, VA 24060, USA*
2. *Department of Chemistry, Lehigh University, Bethlehem, PA 18015, USA*

Experimental Materials and Methods

Materials.

All starting materials and solvents were obtained from commercial sources (Aldrich, Fisher, VWR) and used without further purification, unless otherwise specified.

Powder X-ray Diffraction (PXRD).

A Rigaku Miniflex diffractometer (Cu Kα radiation λ = 1.5418 Å) was used to collect PXRD patterns of prepared MOF powders to determine crystallinity and phase purity. Samples were analyzed over 2θ = 2–50° on a Rigaku Si510 sample holder disc.

Scanning Electron Microscopy (SEM).

SEM images of prepared MOF particles were collected on a LEO 1550 field-emission scanning electron microscope (Carl Zeiss, Oberkochen, Germany) at 5.0 kV with a 5.0 to 7.7 mm working distance. Samples were prepared by drop-casting the MOF suspended in ethanol onto cut fluorine-doped tin oxide (FTO) slides, which were affixed to SEM stages with double-sided copper tape. The stages were then painted with carbon-conductive adhesive and sputtering-coated with Pt and Pd.

Attenuated Total Reflectance-Infrared (ATR-IR) Measurements.

ATR-FTIR spectra were obtained using a Varian 670 FT-IR Spectrometer. All spectra were an average of 64 scans for powdered samples and were recorded from 4000 cm^{-1} to 400 cm^{-1} with 2 cm^{-1} resolution.

UV-Vis Diffuse Reflectance Measurements.

Absorption measurements were performed with an Agilent Technologies Cary 5000 UV-Vis-NIR spectrophotometer equipped with a diffuse reflectance accessory. $BaSO_4$ was used for background measurement.

Nanosecond TRIR Setup for Film Measurements.

Transient infrared absorption measurements for sample films were conducted with a Magnitude Instruments inspIRe system. The probe beam was an IR Globar, while the pump source was an in-board diode-pumped solid-state Nd:YAG laser outputting the $2^{nd}/3^{rd}$ harmonic wavelengths (532/355 nm) at desired rep rates (200-12000 Hz, ca. 2 ns pulse length). For MIL-101(Fe), NH_2-MIL-101(Fe) and OH-MIL-101(Fe), the excited-state kinetics were obtained in 10 cm^{-1} increments from 1100 cm^{-1} to 1800 cm^{-1}, with 6000 laser shots iterated for 50 times at each wavenumber and a time window of 131 μs. For NO_2-MIL-101(Fe), the excited-state kinetics were obtained in 10 cm^{-1} increments from 1100 cm^{-1} to 1800 cm^{-1}, with 1800 laser shots iterated for 50 times at each wavenumber and a time window of 524 μs. A total of 2 kinetic sweeps over the range of 1100-1800 cm^{-1} nm were averaged. The pump beam had an area of 1 cm^2 and the pulse energy was ca. 80/250 μJ for 355/532 nm output. The sample was rotated periodically throughout the measurements to minimize degradation (see Figure S11 for degradation test upon laser exposure). Measurements were conducted under a flow of dry air.

Nanosecond Visible Transient Absorption (VisTA) Setup for Film Measurements.

VisTA measurements for sample films were conducted on a Magnitude Instruments enVISion system. For all measurements, the probe beam was generated by a 150 W Xe arc lamp operated in continuous wave mode. For MIL-101(Fe), the pump source was a Magnitude Instruments tunable OPO outputting 355 nm at 50 Hz (ca. 2 ns pulse length). For NH_2-MIL-101(Fe) and NO_2-MIL-101(Fe), the pump source was an in-board diode-pumped solid-state Nd:YAG laser outputting the $2^{nd}/3^{rd}$ harmonic wavelengths (532/355 nm) at desired rep rates (200-6000 Hz, ca. 2 ns pulse length). The pump beam

was focused to a 1 cm^2 area, and the pulse energy was ~800/133/172 μJ for 355(OPO)/355/532 nm output. Measurements were conducted under a flow of dry air. The excited-state kinetics were obtained in 5 nm increments from 450-700 nm, with 2000 [MIL-101(Fe)]/6000 [NH_2-MIL-101(Fe)]/1800 [NO_2-MIL-101(Fe)] laser shots at each wavelength and a time window of 131 μs [(524 μs for NO_2-MIL-101(Fe)]. A total of 2-3 kinetic sweeps from 450-700 nm were averaged. The TA spectra were generated by averaging specific time points in the kinetic traces from 450-700 nm. The TA spectra were all subjected to a 3-point smooth (moving average) across the wavelengths. The kinetic traces in Figure 2 (main text) were obtained by averaging the kinetics across a 50 nm window at the spectral regions with maximum TA signals. The spectra and kinetics were processed with MATLAB and the kinetics were fit with exponential models in Origin data analysis software.

MIL-101(Fe) Synthesis.

In a typical synthesis of MIL-101(Fe), 2.45 mmol iron chloride hexahydrate ($FeCl_3$•$6H_2O$) and 1.24 mmol benzene-1,4-dicarboxylic acid (BDC) were added to 15 mL *N*,*N*-Dimethylformamide (DMF) and the mixture was heated at 110 °C for 20 h in a Teflon-lined stainless steel Parr bomb. The synthesized brown solid was recovered by centrifugation, washed with ethanol (60 °C for 3 h, 2 times) and then dried in a vacuum oven at 60 °C for 12 hours.

NH_2-MIL-101(Fe) Synthesis.

By using 1.24 mmol of 2-aminobenzene-1,4-dicarboxylic acid (NH_2-BDC) instead of 1.24 mmol benzene-1,4-dicarboxylic acid (BDC), the rest of the synthesis procedure of NH_2-MIL-101(Fe) was the same as that of MIL-101(Fe).

NO_2-MIL-101(Fe) Synthesis.

By using 1.24 mmol of 2-nitrobenzene-1,4-dicarboxylic acid (NO_2-BDC) instead of 1.24 mmol benzene-1,4-dicarboxylic acid (BDC), the rest of the synthesis procedure of NO_2-MIL-101(Fe) was the same as that of MIL-101(Fe).

OH-MIL-101(Fe) Synthesis.

By using 1.24 mmol of 2-hydroxybenzene-1,4-dicarboxylic acid (OH-BDC) instead of 1.24 mmol benzene-1,4-dicarboxylic acid (BDC), the rest of the synthesis procedure of OH-MIL-101(Fe) was the same as that of MIL-101(Fe).

MOF Spin-coating procedure for transient absorption measurements.

A MOF suspension was prepared by dispersing the synthesized particles in ethanol (6% w/w). Prior to spin coating, the CaF_2 substrates (diameter = 25.4 mm, 1 mm thick, Crystran Ltd.) were cleaned with O_2 plasma for 15 min. The MOF film was prepared by spin-coating the alcoholic suspensions of MOF onto the substrate at 1500 rpm to 4500 rpm for 30 s. The volume of the suspension used in each spin coating was 100 μL. The spin-coating process was repeated for 1 more time to obtain thicker films. The films were dried at 100 °C for 10 min after each deposition in N_2 atmosphere.

Density Functional Theory.

All calculations were performed on a cluster that includes a node and the first surrounding ligands in Gaussian16[1] using the M06L[2] functional with LANL2DZ[3-5] basis set on all atoms. Closed shell (R-MIL-101(Fe)) and open shell reduced (R-MIL-101(Fe)$^{-}$) clusters were fully optimized and confirmed as minima with frequency calculations. Counterpoise[6,7] calculations, reduced the basis set superposition error (BSSE) that was apparent when calculating the interaction energy between the node and the ligands. In addition, node and ligand fragment Counterpoise calculations were carried out on the closed shell optimized structure with an electron on the node and on the ligand system (LMCT) to determine the node-ligand binding free energies for all the MOFs. All energies reported are thermally corrected Gibbs Free Energies at 298.15 K.

Supplementary Data

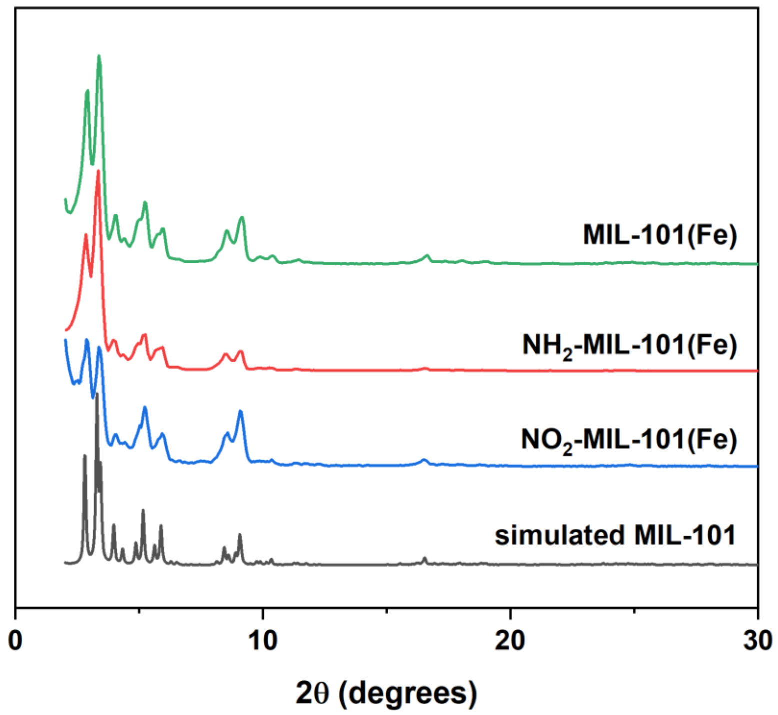

Figure S 1. PXRD of synthesized MIL-101(Fe), NH_2-MIL-101(Fe), NO_2-MIL-101(Fe) and simulated pattern of MIL-101 structure.

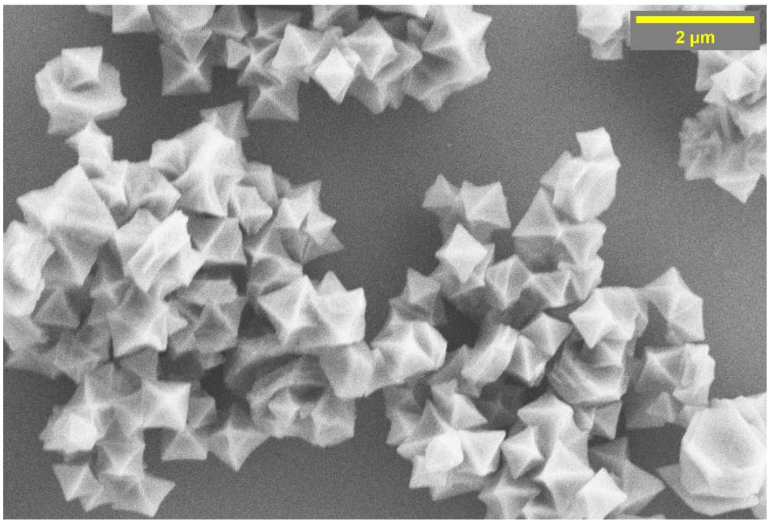

Figure S 2. SEM image of synthesized MIL-101(Fe) microcrystals.

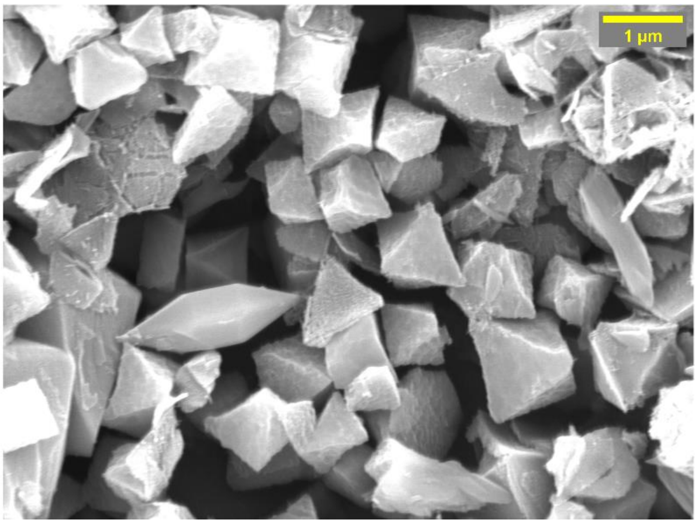

Figure S 3. SEM image of synthesized NH_2-MIL-101(Fe) microcrystals.

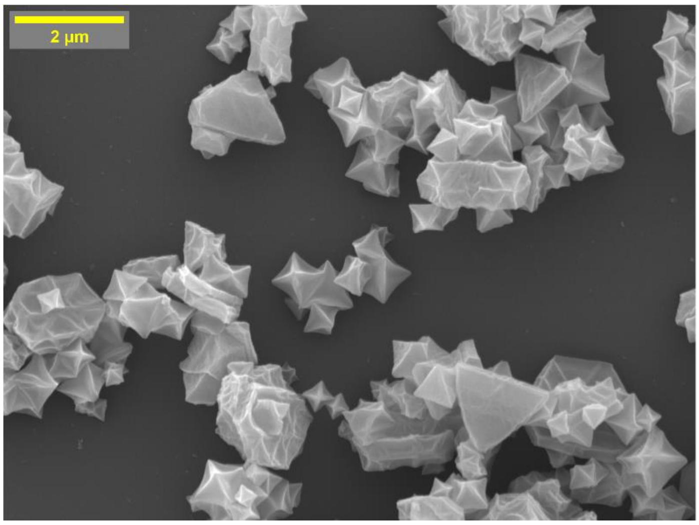

Figure S 4. SEM image of synthesized NO_2-MIL-101(Fe) microcrystals.

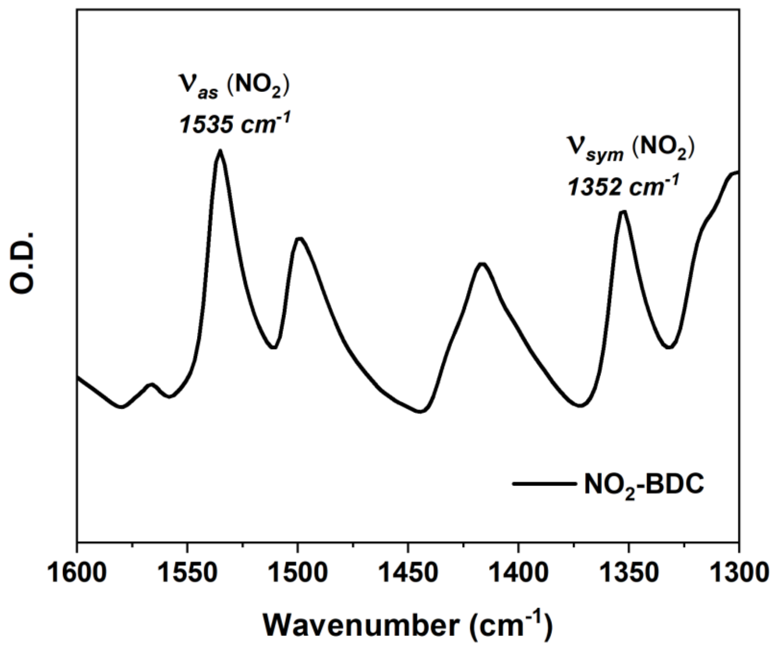

Figure S 5. FTIR spectrum of NO_2-BDC used for NO_2-MIL-101(Fe) synthesis. The absorption peaks corresponding to the asymmetric and symmetric vibrational modes of -NO_2 are found at 1535 cm^{-1} and 1352 cm^{-1}, respectively.

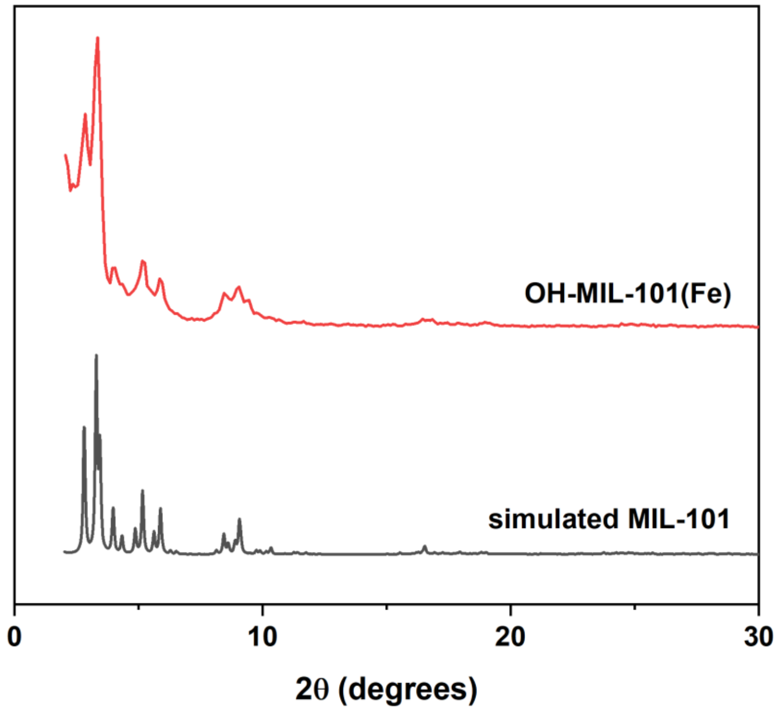

Figure S 6. PXRD of synthesized OH-MIL-101(Fe) and simulated pattern of MIL-101 structure.

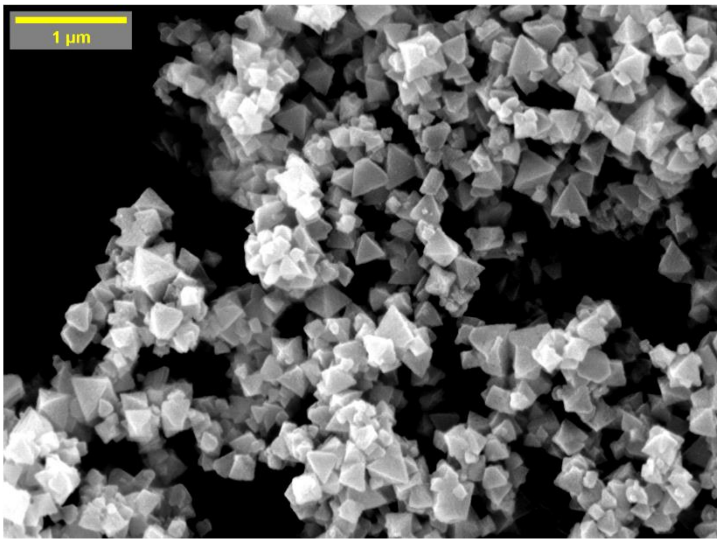

Figure S 7. SEM image of synthesized OH-MIL-101(Fe) microcrystals.

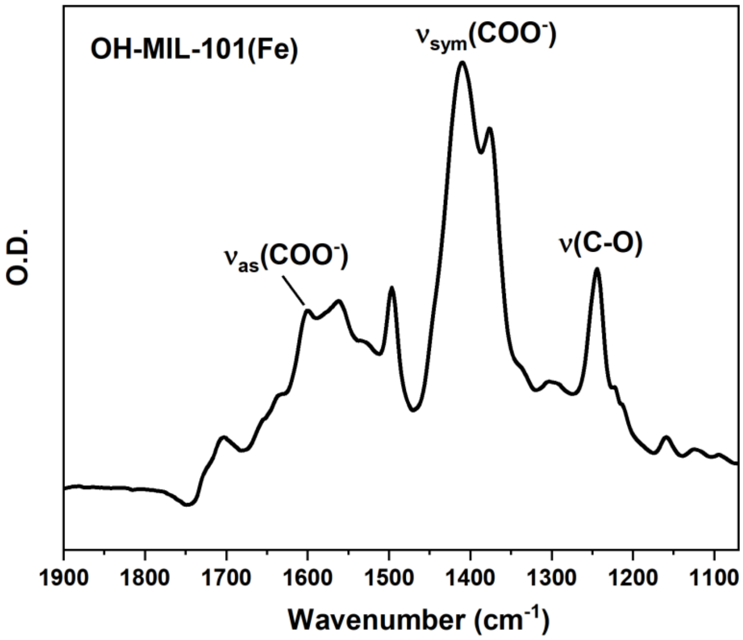

Figure S 8. FTIR absorption spectrum of OH-MIL-101(Fe).

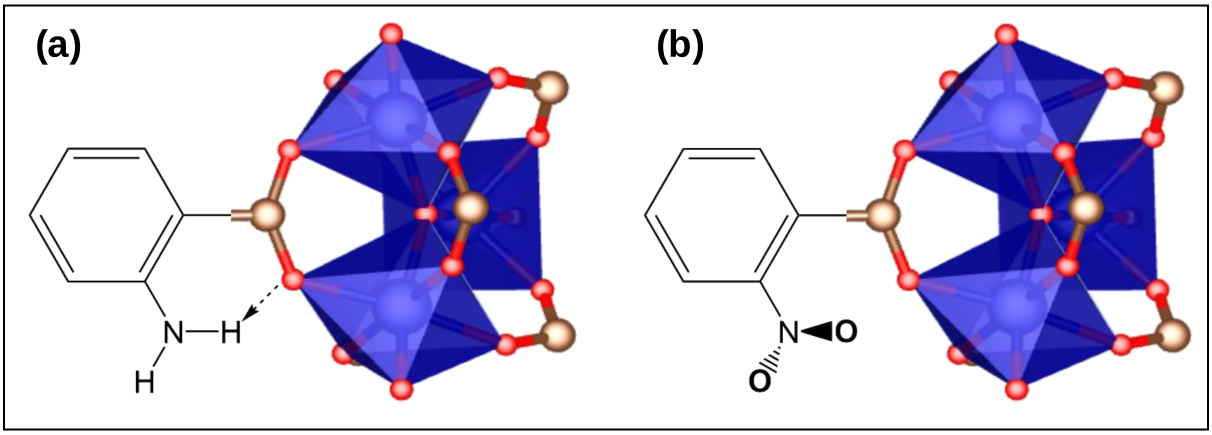

Figure S 9. Ligand R-group node interactions. (a) -NH_2 shows a H-bond to the closest node O and (b) -NO_2 rotates out of plane due to repulsion between -NO_2 and closest node O.

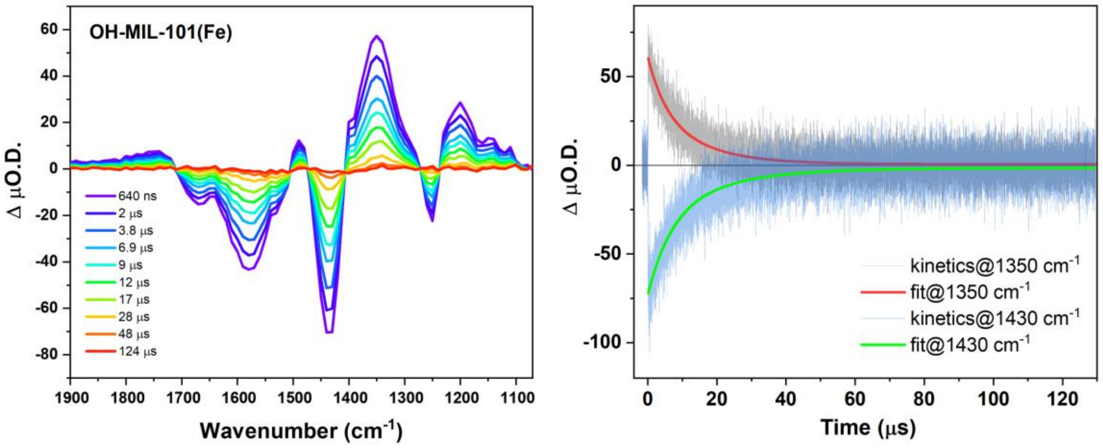

Figure S 10. (Left) TRIR difference absorption spectra at varied time delays upon 355 nm excitation and (Right) representative kinetics with biexponential fits for OH-MIL-101(Fe). at 1430 cm^{-1} where the maximum GSB was recorded, the photoexcited OH-MIL-101(Fe) exhibited a biexponential TRIR decay kinetics with the longer time constant of 20.0 ± 2.7 μs which was close to that of MIL-101(Fe) and the shorter one of 7.0 ± 0.7 μs which was indicative of the attractive interaction between –OH and COO^- in the MOF.

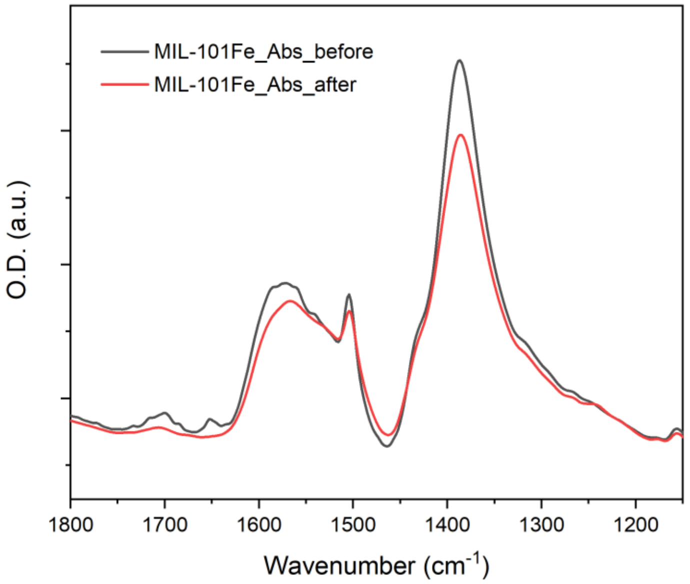

Figure S 11. FTIR spectra of MIL-101(Fe) before and after a 2-hour exposure to 355 nm laser (2 ns pulse, 80 µJ/cm^2 per pulse, 6000 Hz). Upon 2-hour intensive exposure to high energy laser, MIL-101(Fe) tends to exhibit noticeable change in absorbance intensity. The proportion of degradation is ca. 16%, which was evaluated by comparing the OD intensity corresponding to MOF's signature sym(COO^-) mode at 1388 cm^{-1} before/after the laser exposure. Such degradation could result from excited-state evolution pathways in addition to the BET trajectory mainly discussed in the manuscript. Note that for a typical kinetics measurement at a specific wavenumber, with the laser parameters described above, a 10-minute scan is sufficient to obtain data yielding consistent lifetimes as shown in the manuscript – which grants more credibility to the data, as the delta OD signals and their kinetic fits in this report are contributed by more than 98% of undegraded MOFs (estimated from the degradation analysis above for a 10-minute scan). Moreover, to minimize the negative influence of photodegradation, we rotated the samples periodically throughout the measurements so that the data acquired should represent MOFs with minimal/negligible degradation.

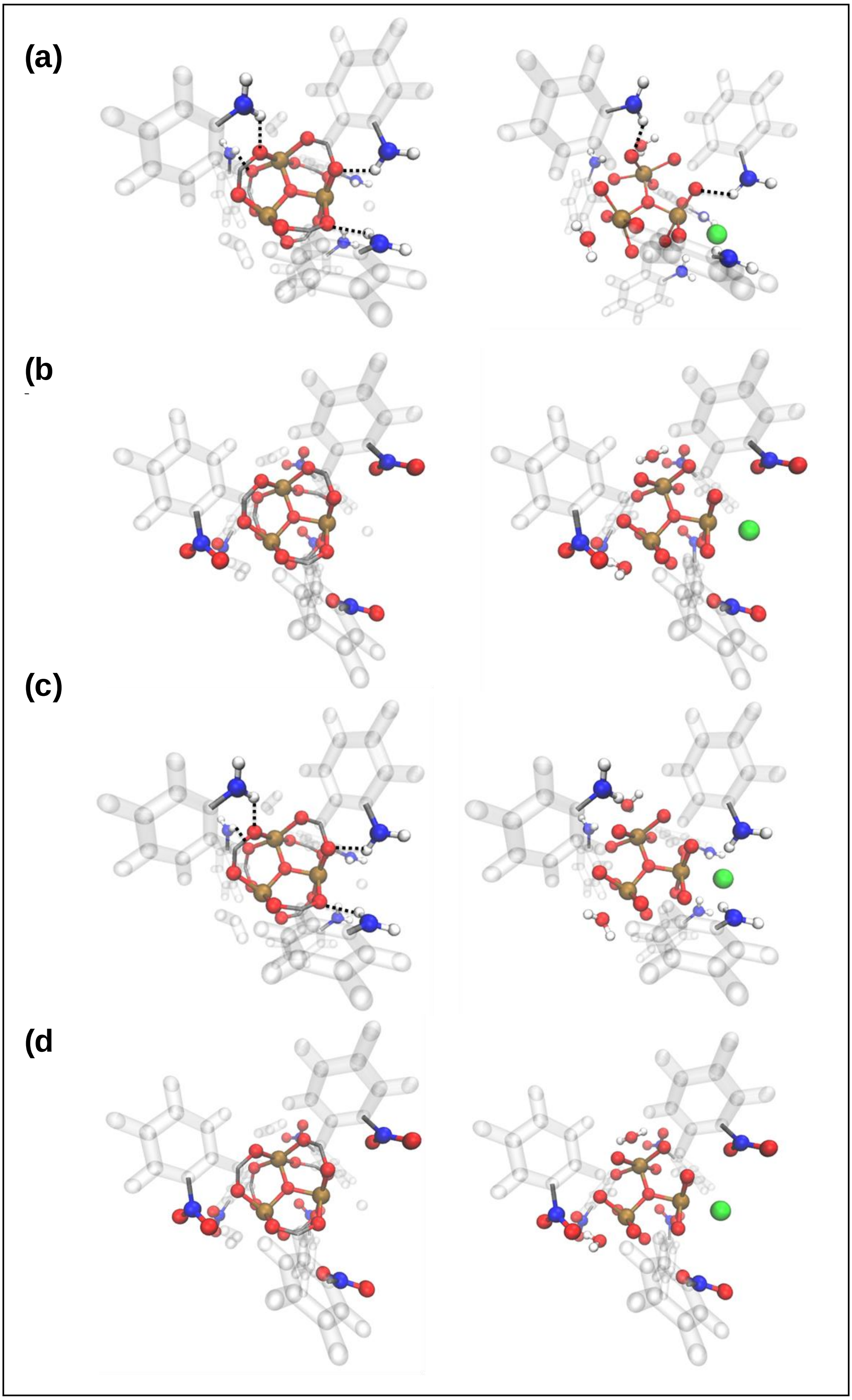

Figure S 12. Fully optimized geometries of (a) NH_2-MIL-101(Fe), (b) NO_2-MIL-101(Fe), (c) NH_2-MIL-101(Fe)$^{-}$, (d) NO_2-MIL-101(Fe)$^{-}$. Waters and Cl^{-} are omitted in the left structures for clarity and the nodes and closet coordination shell are shown in color for emphasis.

Table S1. Ligand R-Group Node Binding Interactions in fully optimized charge transfer structures. M06L/LANL2DZ.

	Average distance (Å)
NH_2-MIL-101(Fe)[,a]	1.918
NO_2-MIL-101(Fe)[,b]	2.772
NH_2-MIL-101(Fe)[–,a]	1.921
NO_2-MIL-101(Fe)[–,b]	2.752

[a]node O—ligand H distance shown in Figure S9
[b]node O—closest ligand O distance shown in Figure S9

Table S2. Calculated Binding Energies of Electron Transfer

	Ligand-Node Binding Energy (kcal/mol)
MIL-101(Fe)	-3631.60
NH_2-MIL-101(Fe)	-3635.36
NO_2-MIL-101(Fe)	-3520.77
MIL-101(Fe)$^{-}$	-2847.85
NH_2-MIL-101(Fe)$^{-}$	-2876.73
NO_2-MIL-101(Fe)$^{-}$	-2744.37

XYZ Coordinates
NH_2-MIL-101(Fe)
107

Fe	0.056002171338	-1.609464602271	0.224619674120
O	0.058113619170	0.210289232390	-0.093950033679
Fe	-0.019794116853	1.348873691743	1.288203352253
O	-1.655038218141	2.158596207332	0.663004253628
C	-1.815088875209	2.656196552286	-0.538769345220
C	-3.000090213352	3.481251557720	-0.800986096926
C	-3.101353908486	4.136215417768	-2.049570432648
C	-4.198994142857	4.922625588770	-2.383332557528
C	-5.245948343635	5.060058139260	-1.446736872793
C	-5.179207750639	4.427498666208	-0.210965547906
C	-4.060246148132	3.628389799115	0.150610982779
H	-6.112736946962	5.665489870271	-1.688199089957
H	-4.249237051951	5.422554181354	-3.342484217801
H	-2.277319123937	4.014310236921	-2.743433347790
O	-0.968123457514	2.441294865566	-1.504989114236

Fe	0.145722449151	0.841580123659	-1.770623213774
O	1.789841225485	1.803697844228	-1.438350838416
C	1.917444566753	2.663441207442	-0.470324228968
O	1.058639467277	2.756829280252	0.515491864298
C	3.072730809890	3.567205896428	-0.482408216183
C	3.258181486459	4.589784613176	0.501246151511
C	4.425733480083	5.393892845959	0.417254754833
C	5.363262868094	5.200075924300	-0.592399848141
C	5.175856096924	4.197952473825	-1.567462987182
C	4.037415312326	3.400401753434	-1.501933569829
H	3.855460606970	2.618042576223	-2.229515698569
H	6.244754557864	5.831278349034	-0.626726775911
H	4.576118469046	6.171734046533	1.160285115400
O	1.208186967249	-0.561564941151	-2.568759430848
C	1.933570162406	-1.448020540028	-1.961046414206
O	1.673431752887	-1.877395758057	-0.744307248070
C	3.088135671965	-2.018930921409	-2.648790129021
C	3.564086313198	-1.343894284727	-3.797426815766
C	4.647891478040	-1.820144284815	-4.525625361693
C	5.278844576462	-3.011663306444	-4.103715935557
C	4.834031222996	-3.695231662925	-2.978666810572
C	3.732916183614	-3.221194361780	-2.211032124872
H	6.125512381873	-3.400457350389	-4.659215050754
H	5.005651744519	-1.287088080101	-5.397485630547
H	3.051617069660	-0.431329869103	-4.081488691465
O	-1.494150390757	-0.056662689437	-2.333162924568
C	-1.765551041007	-1.295080510331	-2.056173866762
O	-0.997152484704	-2.048393381560	-1.295366776054
C	-2.962958681539	-1.914542130246	-2.618845252626
C	-3.823568649645	-1.101077029098	-3.395694554096
C	-4.987414192181	-1.599687250945	-3.966036901761
C	-5.314817469260	-2.960012710742	-3.764685733557
C	-4.494171463172	-3.782990721764	-3.006203306889
C	-3.297411768107	-3.294160339694	-2.405256353420
H	-6.218148718984	-3.369453965819	-4.203637173540
H	-5.635636963777	-0.958234588256	-4.549540084765
H	-3.542795742155	-0.061267140149	-3.517983532559
O	-1.142703811105	0.311886645727	2.512195732585
C	-1.870778207154	-0.726887260140	2.218502121864
O	-1.581744642790	-1.533434198500	1.227491824271
C	-3.062110009587	-1.022310456811	3.013601431020
C	-3.806989774857	-2.238298239328	2.861002819646
C	-4.952874077379	-2.421350165604	3.687563865001
C	-5.343616708501	-1.459270517957	4.608858009173
C	-4.609762554378	-0.260650044618	4.758444751334
C	-3.486533467926	-0.063936272685	3.964337517720
H	-2.896901896655	0.842738326749	4.053660272429
H	-4.916078450593	0.486056430100	5.480370870592
H	-6.223244418297	-1.634126223609	5.218594273101
O	1.616498545703	0.692609635362	2.121296902097
C	1.879763566801	-0.571452319531	2.279351964967
O	1.095324671898	-1.521768945053	1.817698842352
C	3.073528099198	-0.971943017612	3.014831583787
C	3.399929770229	-2.346537081488	3.271537120488
C	4.594456586267	-2.615242711524	4.002603083305

C	5.421871402866	-1.592099037270	4.442037650470
C	5.105292430551	-0.239793545356	4.175592548563
C	3.943887297601	0.048405023724	3.471880654545
H	3.677073245663	1.071906457391	3.233526425811
H	5.762128614260	0.554457588506	4.506818342554
H	6.324192556289	-1.837473578675	4.991391516873
Cl	0.048385814485	-4.012938657175	0.633008771581
O	0.131790096431	1.599638523380	-3.641394229646
H	-0.587293167847	2.263690244478	-3.627103336198
H	-0.038723871875	0.888562812078	-4.286675721044
O	-0.000287337250	2.584361404552	2.942073700456
H	-0.579462995167	2.091527879998	3.562169520748
H	0.931788965354	2.518884336611	3.240240171698
H	-4.756003500360	-4.826324141609	-2.856812624737
H	4.850159477476	-3.650353416858	4.208073146849
H	5.329638582595	-4.608487710430	-2.663329575280
H	5.907273189855	4.050457761970	-2.352114075802
H	-5.988931027167	4.538679444098	0.503870594558
H	-5.523833733660	-3.338683120521	3.581887531839
N	-2.532457566547	-4.156946229997	-1.669109645616
N	3.325849965934	-3.932904568072	-1.114290664254
N	2.628774914516	-3.397521314334	2.862191026227
N	2.348817470115	4.825801882234	1.508664748216
N	-3.463070083133	-3.220445184168	1.974498233543
N	-4.040398108288	3.017090147220	1.379072471193
H	2.476012593504	5.618030818762	2.116483926252
H	1.482116883761	4.301443138160	1.516863498719
H	-3.236104153056	2.456741710867	1.633146150119
H	-4.838715816258	3.080408373468	1.988486199451
H	2.912166171081	-4.337403272410	3.088259480318
H	1.777292524494	-3.258416175990	2.324135843277
H	3.759109285200	-4.817226133849	-0.905814578340
H	2.546244557507	-3.618888537227	-0.547064650472
H	-1.670168510317	-3.857648807494	-1.221413096195
H	-2.809296328608	-5.122664644695	-1.593981457590
H	-2.643641302063	-3.120758172167	1.386078450194
H	-3.992019607557	-4.076498402636	1.946811053994

NH_2-MIL-101(Fe)$^{-}$
107

Fe	0.056002290846	-1.609464288680	0.224619749882
O	0.058113663266	0.210289617916	-0.093949914972
Fe	-0.019794112238	1.348873925642	1.288203540852
O	-1.655038250084	2.158596348070	0.663004430981
C	-1.815088956499	2.656196633574	-0.538769193918
C	-3.000090442799	3.481251421433	-0.800985980074
C	-3.101354254078	4.136215199759	-2.049570346316
C	-4.198994662638	4.922625099273	-2.383332538830
C	-5.245948922391	5.060057446104	-1.446736890283
C	-5.179208207208	4.427498059219	-0.210965527190
C	-4.060246419206	3.628389482359	0.150611076486
H	-6.112737664074	5.665488961196	-1.688199155867
H	-4.249237660468	5.422553631584	-3.342484225561
H	-2.277319425200	4.014310169941	-2.743433232544
O	-0.968123508648	2.441295072233	-1.504988952924

Fe	0.145722504444	0.841580423123	-1.770623101438
O	1.789841277955	1.803698142454	-1.438350637979
C	1.917444626142	2.663441426468	-0.470323968321
O	1.058639461097	2.756829550285	0.515492068074
C	3.072731006334	3.567205958365	-0.482407831830
C	3.258181735030	4.589784693996	0.501246504110
C	4.425733852345	5.393892754339	0.417255185722
C	5.363263322174	5.200075635460	-0.592399304934
C	5.175856509845	4.197952157883	-1.567462406820
C	4.037415593139	3.400401617152	-1.501933073333
H	3.855460836348	2.618042437300	-2.229515186504
H	6.244755109101	5.831277926662	-0.626726168287
H	4.576118877078	6.171733972146	1.160285519843
O	1.208187072963	-0.561564595749	-2.568759364354
C	1.933570190186	-1.448020303381	-1.961046420466
O	1.673431886741	-1.877395396787	-0.744307175536
C	3.088135484311	-2.018930993404	-2.648790243269
C	3.564086171299	-1.343894481038	-3.797426986417
C	4.647891117137	-1.820144764075	-4.525625667483
C	5.278843943475	-3.011663955858	-4.103716323176
C	4.834030551142	-3.695232194861	-2.978667146924
C	3.732915740597	-3.221194610266	-2.211032306372
H	6.125511572323	-3.400458225496	-4.659215547869
H	5.005651416046	-1.287088654496	-5.397485980990
H	3.051617146899	-0.431329921373	-4.081488795587
O	-1.494150333662	-0.056662435116	-2.333162792844
C	-1.765550918423	-1.295080270861	-2.056173796617
O	-0.997152322880	-2.048393139953	-1.295366707507
C	-2.962958476246	-1.914542025565	-2.618845201802
C	-3.823568555205	-1.101077054640	-3.395694515903
C	-4.987414022888	-1.599687455089	-3.966036857599
C	-5.314817102695	-2.960012960691	-3.764685666122
C	-4.494170976064	-3.782990841013	-3.006203222877
C	-3.297411357427	-3.294160267178	-2.405256276712
H	-6.218148288670	-3.369454358541	-4.203637105368
H	-5.635636885416	-0.958234898592	-4.549540055426
H	-3.542795800024	-0.061267125465	-3.517983499309
O	-1.142703752942	0.311886790379	2.512195913522
C	-1.870778183811	-0.726887063395	2.218502193365
O	-1.581744567083	-1.533433941842	1.227491839823
C	-3.062110025899	-1.022310319233	3.013601416237
C	-3.806989963230	-2.238297976975	2.861002539302
C	-4.952874290210	-2.421349922476	3.687563549030
C	-5.343616800519	-1.459270424674	4.608857896606
C	-4.609762482227	-0.260650083677	4.758444899330
C	-3.486533368589	-0.063936290710	3.964337719107
H	-2.896901672546	0.842738206239	4.053660689587
H	-4.916078264265	0.486056269094	5.480371192896
H	-6.223244531617	-1.634126144050	5.218594124310
O	1.616498606983	0.692609885489	2.121297016404
C	1.879763679028	-0.571452057195	2.279352029403
O	1.095324786345	-1.521768709558	1.817698901512
C	3.073528271156	-0.971942788068	3.014831525215
C	3.399929958457	-2.346536870369	3.271536920825
C	4.594456861696	-2.615242560880	4.002602720632

C	5.421871745649	-1.592098921743	4.442037239128
C	5.105292764996	-0.239793406746	4.175592250185
C	3.943887543382	0.048405223995	3.471880527698
H	3.677073464153	1.071906672861	3.233526374682
H	5.762129014787	0.554457696547	4.506817986259
H	6.324192959929	-1.837473506509	4.991390985241
Cl	0.048386023627	-4.012938537599	0.633008801355
O	0.131790165278	1.599638809275	-3.641394106987
H	-0.587293398472	2.263690202679	-3.627103422044
H	-0.038723274001	0.888563030449	-4.286675660812
O	-0.000287302175	2.584361580117	2.942073897067
H	-0.579462709800	2.091527958415	3.562169867128
H	0.931789076668	2.518884840865	3.240240191047
H	-4.756002853412	-4.826324301155	-2.856812527773
H	4.850159756159	-3.650353281002	4.208072705495
H	5.329637704547	-4.608488376232	-2.663329979593
H	5.907273663441	4.050457295677	-2.352113410490
H	-5.988931512984	4.538678700284	0.503870593870
H	-5.523834067276	-3.338682779269	3.581887012051
N	-2.532456990554	-4.156946005335	-1.669109552050
N	3.325849487029	-3.932904736183	-1.114290802580
N	2.628774994957	-3.397521044568	2.862190885766
N	2.348817647389	4.825802122500	1.508665005709
N	-3.463070441746	-3.220444789044	1.974497737279
N	-4.040398223499	3.017089959830	1.379072623626
H	2.476012774812	5.618031107686	2.116484119411
H	1.482116986025	4.301443501142	1.516863670802
H	-3.236104083747	2.456741833861	1.633146398440
H	-4.838715959700	3.080408000280	1.988486335024
H	2.912166262895	-4.337403030054	3.088259213547
H	1.777292530225	-3.258415839199	2.324135803564
H	3.759108617535	-4.817226413497	-0.905814799590
H	2.546244236463	-3.618888532263	-0.547064640720
H	-1.670167932244	-3.857648440247	-1.221413098252
H	-2.809295553672	-5.122664477433	-1.593981360440
H	-2.643641659734	-3.120757775038	1.386077945359
H	-3.992020150789	-4.076497887213	1.946810322992

NO_2-MIL-101(Fe)
107

Fe	0.416341000000	-1.718503000000	0.640615000000
O	0.219366000000	-0.010592000000	-0.177209000000
Fe	0.294001000000	1.534714000000	0.820294000000
O	-1.464120000000	2.135434000000	0.311446000000
C	-1.870462000000	2.251446000000	-0.903508000000
C	-3.143272000000	2.990727000000	-1.119859000000
C	-4.091106000000	2.436246000000	-1.993016000000
C	-5.358604000000	3.017949000000	-2.131708000000
C	-5.699138000000	4.158060000000	-1.387482000000
C	-4.768296000000	4.725460000000	-0.509458000000
C	-3.503152000000	4.142358000000	-0.395032000000
H	-6.679896000000	4.604204000000	-1.490238000000
H	-6.078985000000	2.572942000000	-2.806011000000
H	-3.833788000000	1.527117000000	-2.524890000000
O	-1.302424000000	1.726233000000	-1.938749000000

Fe	-0.163381000000	0.120269000000	-1.957774000000
O	1.415212000000	1.194376000000	-2.366993000000
C	1.696981000000	2.255183000000	-1.683550000000
O	1.120268000000	2.614772000000	-0.588177000000
C	2.745577000000	3.162368000000	-2.217612000000
C	3.232964000000	4.263099000000	-1.490427000000
C	4.162916000000	5.155390000000	-2.024178000000
C	4.649457000000	4.930469000000	-3.318960000000
C	4.193091000000	3.830989000000	-4.062201000000
C	3.243695000000	2.960923000000	-3.516427000000
H	2.852070000000	2.120424000000	-4.076585000000
H	5.376126000000	5.612625000000	-3.741475000000
H	4.499817000000	6.000757000000	-1.437712000000
O	0.846746000000	-1.430578000000	-2.515452000000
C	1.770223000000	-2.047506000000	-1.851086000000
O	1.875409000000	-2.123448000000	-0.567773000000
C	2.890266000000	-2.622790000000	-2.647202000000
C	3.486878000000	-1.816558000000	-3.628495000000
C	4.640073000000	-2.250302000000	-4.296751000000
C	5.213568000000	-3.492509000000	-3.983879000000
C	4.615747000000	-4.319072000000	-3.023918000000
C	3.456772000000	-3.879024000000	-2.382410000000
H	6.108601000000	-3.824602000000	-4.494818000000
H	5.093084000000	-1.620613000000	-5.052730000000
H	3.039113000000	-0.851348000000	-3.837823000000
O	-1.778263000000	-0.889009000000	-1.770769000000
C	-1.797707000000	-2.084394000000	-1.244445000000
O	-0.889814000000	-2.606659000000	-0.513969000000
C	-3.033902000000	-2.872922000000	-1.471790000000
C	-3.429042000000	-3.799093000000	-0.498172000000
C	-4.679105000000	-4.425927000000	-0.587097000000
C	-5.556464000000	-4.115260000000	-1.636944000000
C	-5.176086000000	-3.199270000000	-2.627039000000
C	-3.911500000000	-2.614162000000	-2.540874000000
H	-6.529750000000	-4.586095000000	-1.691207000000
H	-4.978557000000	-5.129208000000	0.178760000000
H	-2.753898000000	-3.980882000000	0.331149000000
O	-0.535254000000	0.809901000000	2.399599000000
C	-1.246713000000	-0.267624000000	2.480096000000
O	-1.075967000000	-1.350486000000	1.807033000000
C	-2.323477000000	-0.280976000000	3.508182000000
C	-3.594670000000	-0.842832000000	3.289361000000
C	-4.535681000000	-0.968347000000	4.317103000000
C	-4.213914000000	-0.496412000000	5.593436000000
C	-2.965040000000	0.100762000000	5.824143000000
C	-2.026262000000	0.204602000000	4.789788000000
H	-1.046783000000	0.630434000000	4.960793000000
H	-2.716745000000	0.473848000000	6.809743000000
H	-4.935205000000	-0.583590000000	6.395976000000
O	2.031505000000	1.178359000000	1.560678000000
C	2.289655000000	0.043846000000	2.150736000000
O	1.701911000000	-1.074004000000	1.958772000000
C	3.437617000000	0.039508000000	3.098694000000
C	3.465459000000	-0.769049000000	4.248717000000
C	4.612847000000	-0.896953000000	5.034331000000

C	5.758943000000	-0.170819000000	4.687718000000
C	5.737832000000	0.681597000000	3.573647000000
C	4.585447000000	0.781728000000	2.782270000000
H	4.559066000000	1.417318000000	1.905404000000
H	6.617575000000	1.257979000000	3.315545000000
H	6.652697000000	-0.258960000000	5.292335000000
Cl	0.678830000000	-3.741226000000	1.643512000000
O	-0.682650000000	0.230080000000	-3.907420000000
H	-1.283388000000	0.994020000000	-4.010374000000
H	-1.113938000000	-0.603402000000	-4.221878000000
O	0.492107000000	3.280524000000	1.818408000000
H	1.443497000000	3.470142000000	1.971914000000
H	0.040603000000	4.029714000000	1.362212000000
H	-5.823496000000	-2.960525000000	-3.461474000000
H	4.584108000000	-1.541456000000	5.903513000000
H	5.012335000000	-5.295691000000	-2.777890000000
H	4.567041000000	3.662575000000	-5.064007000000
H	-4.996925000000	5.608320000000	0.073317000000
H	-5.497636000000	-1.411625000000	4.095041000000
N	-3.485720000000	-1.748816000000	-3.648547000000
N	2.757200000000	-4.841062000000	-1.499283000000
N	2.238190000000	-1.429832000000	4.754305000000
N	2.776684000000	4.529782000000	-0.102653000000
N	-4.032600000000	-1.246306000000	1.939078000000
N	-2.535722000000	4.814831000000	0.493361000000
O	-4.277857000000	-0.837995000000	-4.061431000000
O	-2.337133000000	-1.977798000000	-4.176757000000
O	-4.913408000000	-2.171274000000	1.851429000000
O	-3.558935000000	-0.623078000000	0.927890000000
O	1.131146000000	-0.807167000000	4.618054000000
O	2.372702000000	-2.537263000000	5.367177000000
O	3.470095000000	-5.693700000000	-0.871406000000
O	1.484175000000	-4.795853000000	-1.482252000000
O	3.252588000000	3.804563000000	0.836308000000
O	1.967032000000	5.493455000000	0.080602000000
O	-1.295473000000	4.794301000000	0.163798000000
O	-2.986283000000	5.412195000000	1.523438000000

NO_2-MIL-101(Fe)$^-$
107

Fe	-0.347215634883	1.747132287012	0.416516578288
O	-0.207293158375	-0.053629171605	-0.185375848026
Fe	-0.217037900300	-1.456990390619	1.005856642020
O	1.502488419867	-2.135350189223	0.463790992066
C	1.826708379622	-2.410643066159	-0.750203815363
C	3.081360764188	-3.184033270406	-0.952944379926
C	3.969473124270	-2.755771619101	-1.950987241210
C	5.224195738653	-3.362859941582	-2.096864924427
C	5.612274936248	-4.401053290449	-1.235912706739
C	4.741426346381	-4.841666460082	-0.232510283684
C	3.487434940139	-4.236335394137	-0.110978165548
H	6.583457038937	-4.866286401308	-1.344788131911
H	5.898502883860	-3.015354486767	-2.869014176883
H	3.678319708303	-1.920131450193	-2.577527951949
O	1.191477999036	-2.017415199467	-1.804762282877

Fe	0.055620449415	-0.416166945338	-1.955537891936
O	-1.548041908829	-1.518483679539	-2.117665575794
C	-1.784839387384	-2.479762670790	-1.285998424752
O	-1.136743436661	-2.701100377492	-0.193879388763
C	-2.867819091032	-3.437797346458	-1.628174450391
C	-3.306903988821	-4.431027650240	-0.734502645694
C	-4.271465544871	-5.375414452348	-1.086124891241
C	-4.843059122597	-5.314151613161	-2.364131608023
C	-4.435998164154	-4.323922597939	-3.271481031227
C	-3.451260471756	-3.400218845120	-2.906379460898
H	-3.096887584652	-2.642625294358	-3.594897436351
H	-5.597117241313	-6.037840526376	-2.646218689870
H	-4.569487282832	-6.134988098273	-0.374561580829
O	-0.987620468520	1.059879661909	-2.640683244626
C	-1.863966264039	1.766145835259	-2.001937513957
O	-1.883187387815	2.007547708192	-0.735020479383
C	-3.033953242385	2.245186138531	-2.789909320499
C	-3.695681852004	1.325311968318	-3.617458555774
C	-4.890382968293	1.680765656998	-4.258473345832
C	-5.440238271134	2.958450275793	-4.071273686529
C	-4.778703703662	3.895730223471	-3.267533041797
C	-3.580027811299	3.530528690796	-2.652551715234
H	-6.366976693795	3.230810348292	-4.560564495905
H	-5.393583766715	0.963534820150	-4.895341243461
H	-3.264035424879	0.336858955562	-3.729637254503
O	1.680584868572	0.592925469339	-2.007313184607
C	1.736534380594	1.845830933665	-1.642020971344
O	0.880035885379	2.466575410955	-0.926680910561
C	2.955742323890	2.586428673800	-2.050295470346
C	3.416078052756	3.626224608438	-1.232509211969
C	4.658167293545	4.224148066298	-1.483883977124
C	5.463138937192	3.772453327767	-2.540502920667
C	5.016444250746	2.740489574001	-3.376879549751
C	3.759730756597	2.183731067480	-3.132659668371
H	6.431195366988	4.222848318685	-2.719185266731
H	5.008898605210	5.017129605525	-0.836596661762
H	2.798000033429	3.919774047197	-0.390815797049
O	0.716624626548	-0.543213197709	2.420249591470
C	1.433154344982	0.528698583554	2.313933370936
O	1.219164120768	1.517612900474	1.519568167439
C	2.576140132147	0.663636445094	3.258355549613
C	3.830566315727	1.180210431455	2.885442543347
C	4.838213175704	1.427658549285	3.824019226685
C	4.601775693416	1.126983246396	5.169000205187
C	3.370370027467	0.576697354054	5.556755827431
C	2.364535501121	0.349870869791	4.608692746306
H	1.398144719524	-0.040820892940	4.897488715334
H	3.187955978742	0.335931022394	6.596470933567
H	5.375118023915	1.309599388180	5.904203078038
O	-1.900834091458	-0.991375242528	1.807174563116
C	-2.117679471865	0.212081853223	2.261706947953
O	-1.542722800263	1.290148794288	1.888771769650
C	-3.199815601153	0.349550082287	3.274891190745
C	-3.149886474011	1.299545130634	4.310317791694
C	-4.242136412775	1.538660716265	5.146895491910

C	-5.409673012232	0.785228758434	4.973273350718
C	-5.463971917441	-0.203569299737	3.979520632050
C	-4.367087890908	-0.415933024510	3.133328418288
H	-4.400040039397	-1.159256780390	2.345961142701
H	-6.359664515962	-0.799722229355	3.856573230545
H	-6.260980698027	0.959149906224	5.619147153899
Cl	-0.539780353829	3.884521447788	1.165246269841
O	0.443493955951	-0.780914135447	-3.904725156536
H	1.035110960201	-1.557594523860	-3.947714275162
H	0.853829937779	0.000935353576	-4.351850151097
O	-0.350177617883	-3.057893667117	2.231708487126
H	-1.289427850818	-3.216870296300	2.470866393363
H	0.068983010745	-3.863922138720	1.847156171819
H	5.606441459850	2.390073448958	-4.214455609585
H	-4.154688831448	2.289294937213	5.921871623216
H	-5.156823598501	4.899717134984	-3.123892021282
H	-4.875767440540	-4.282112325874	-4.259748002703
H	5.007382654103	-5.644429274139	0.442899707498
H	5.783732987407	1.829254919962	3.483611885761
N	3.259949349057	1.187308679140	-4.088909061997
N	-2.821933603731	4.591272568220	-1.949027506595
N	-1.890826561760	2.007825847613	4.644307648284
N	-2.759349354108	-4.521447551026	0.642898720609
N	4.177891780222	1.402347536998	1.468454020317
N	2.580644769912	-4.779451693286	0.918652757267
O	4.021680196568	0.223236760333	-4.432247566796
O	2.078947484073	1.357678855594	-4.565248373531
O	5.051989667237	2.299692479072	1.204264253550
O	3.637060420183	0.658900019838	0.579601810161
O	-0.796085690804	1.362002094085	4.516723099411
O	-1.982818497859	3.186150809879	5.116743660225
O	-3.490321577326	5.524524672356	-1.390692304054
O	-1.550665071220	4.536171583261	-2.010392941746
O	-3.170666458409	-3.676856530077	1.509824182329
O	-1.940422465641	-5.461423527005	0.895069685434
O	1.321193050744	-4.789343285505	0.671804075688
O	3.098229844460	-5.243741810272	1.985165140279

References

[1] Gaussian 16, Revision C.01, Frisch, M. J.; Trucks, G. W.; Schlegel, H. B.; Scuseria, G. E.; Robb, M. A.; Cheeseman, J. R.; Scalmani, G.; Barone, V.; Petersson, G. A.; Nakatsuji, H.; Li, X.; Caricato, M.; Marenich, A. V.; Bloino, J.; Janesko, B. G.; Gomperts, R.; Mennucci, B.; Hratchian, H. P.; Ortiz, J. V.; Izmaylov, A. F.; Sonnenberg, J. L.; Williams-Young, D.; Ding, F.; Lipparini, F.; Egidi, F.; Goings, J.; Peng, B.; Petrone, A.; Henderson, T.; Ranasinghe, D.; Zakrzewski, V. G.; Gao, J.; Rega, N.; Zheng, G.; Liang, W.; Hada, M.; Ehara, M.; Toyota, K.; Fukuda, R.; Hasegawa, J.; Ishida, M.; Nakajima, T.; Honda, Y.; Kitao, O.; Nakai, H.; Vreven, T.; Throssell, K.; Montgomery, J. A., Jr.; Peralta, J. E.; Ogliaro, F.; Bearpark, M. J.; Heyd, J. J.; Brothers, E. N.; Kudin, K. N.; Staroverov, V. N.; Keith, T. A.; Kobayashi, R.; Normand, J.; Raghavachari, K.; Rendell, A. P.; Burant, J. C.; Iyengar, S. S.; Tomasi, J.; Cossi, M.; Millam, J. M.; Klene, M.; Adamo, C.; Cammi, R.; Ochterski, J. W.; Martin, R. L.; Morokuma, K.; Farkas, O.; Foresman, J. B.; Fox, D. J. Gaussian, Inc., Wallingford CT, **2016**.

[2] Zhao Y, Truhlar DG. A new local density functional for main-group thermochemistry, transition metal bonding, thermochemical kinetics, and noncovalent interactions. J Chem Phys. 2006 Nov 21;125(19):194101. doi: 10.1063/1.2370993. PMID: 17129083.

[3] *Methods of Electronic Structure Theory*; Schaefer, H. F., Ed.; Springer US: Boston, MA, **1977**. https://doi.org/10.1007/978-1-4757-0887-5.

[4] Hay, P. J.; Wadt, W. R. Ab Initio Effective Core Potentials for Molecular Calculations. Potentials for K to Au Including the Outermost Core Orbitals. *The Journal of Chemical Physics* **1985**, *82* (1), 299–310. https://doi.org/10.1063/1.448975.

[5] Wadt, W. R.; Hay, P. J. Ab Initio Effective Core Potentials for Molecular Calculations. Potentials for Main Group Elements Na to Bi. *The Journal of Chemical Physics* **1985**, *82* (1), 284–298. https://doi.org/10.1063/1.448800.

[6] S. Simon, M. Duran, and J. J. Dannenberg, “How does basis set superposition error change the potential surfaces for hydrogen bonded dimers?,” *J. Chem. Phys.*, **105** (1996) 11024-31. DOI: 10.1063/1.472902

[7] S. F. Boys and F. Bernardi, “Calculation of Small Molecular Interactions by Differences of Separate Total Energies – Some Procedures with Reduced Errors,” *Mol. Phys.*, **19** (1970) 553. DOI: 10.1080/00268977000101561
